# Atypical Presentation of Marantic Endocarditis

**DOI:** 10.14797/mdcvj.1105

**Published:** 2022-07-11

**Authors:** Nina Manian, Priya Arunachalam, Lamees Ibrahim El Nihum, Qasim Al Abri, Roberto Barrios, Mohammed Chamsi-Pasha, Mahesh Ramchandani

**Affiliations:** 1 Texas A&M College of Medicine, Bryan, Texas, US; 2 Methodist DeBakey Heart & Vascular Center, Houston Methodist Hospital, Houston, Texas, US; 3 Houston Methodist Hospital, Houston, Texas, US

**Keywords:** marantic endocarditis, aortic valve, aortic stenosis, valve replacement, antiphospholipid syndrome

## Abstract

We describe a 39-year-old man referred for surgical aortic valve replacement for severe symptomatic aortic stenosis. Intraoperative inspection was unexpectedly consistent with marantic endocarditis. Pathology confirmed nonbacterial thrombotic endocarditis. We present high-resolution intraoperative, diagnostic, and pathology images of nonbacterial thrombotic endocarditis in a patient with antiphospholipid syndrome with atypical presentation.

## Case presentation

**Figure 1:**
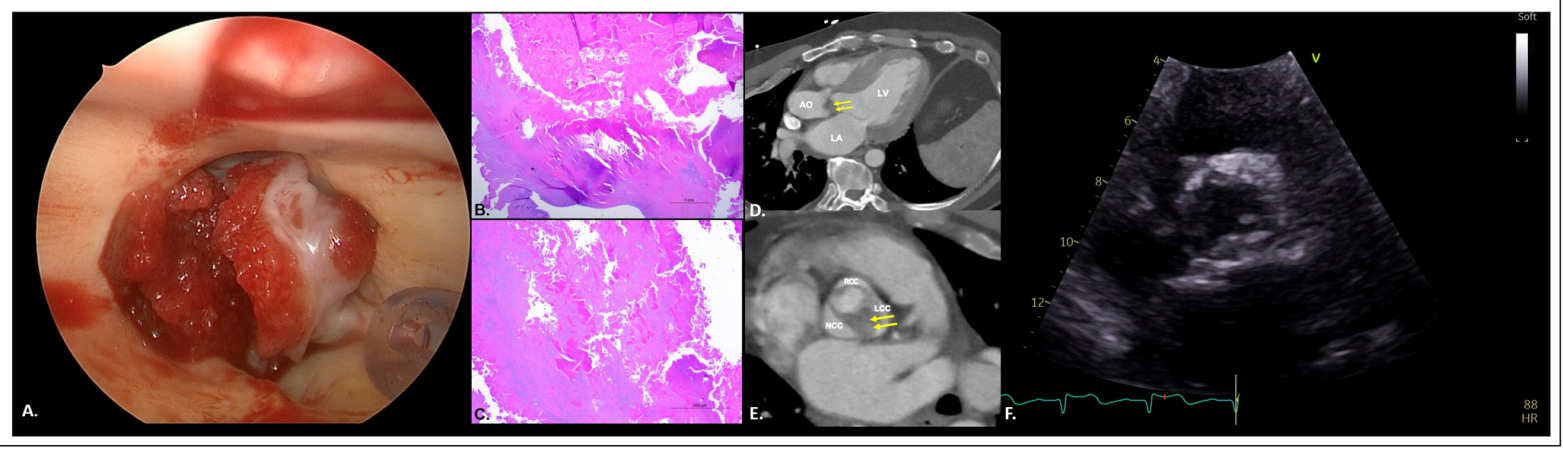
Marantic endocarditis. **(A)** Intraoperatively, the valve appears stenotic with a heavily calcified annulus and fresh-formed thrombus on both the outflow and inflow. **(B)** Low magnification of a vegetation composed of fibrin, forming an eosinophilic mass. Mature fibrosis is seen at the base of the vegetation. **(C)** High magnification shows that vegetation is composed predominantly of fibrin and red blood cells. **(D)** 3-chamber view on non-gated computed tomography shows ventricular hypertrophy and subvalvular aortic valve thickening extending to the left ventricular outflow tract (arrows). **(E)** Short axis of the aortic valve on non-gated computed tomography shows bicuspid aortic valve opening (Sievers type 0) with severe thickening of the left coronary cusp leaflet and no calcification (arrows). **(F)** Parasternal short axis view shows thickened aortic valve leaflets with systolic doming and restricted opening.

A 39-year-old man was referred to our institution due to progressively worsening shortness of breath for the past 6 months. He denied chest pain, palpitations, and syncope. Coronary angiography revealed normal coronary arteries. Echocardiography demonstrated a stenotic bicuspid aortic valve with a mean gradient of 84 mm Hg. We elected to proceed with minimally invasive surgical aortic valve replacement (SAVR) via right mini-thoracotomy. Following cardiopulmonary bypass (CPB) and cardioplegic diastolic arrest, aortotomy was performed and the aortic valve was visualized. The valve appeared stenotic with a heavily calcified annulus and fresh-formed thrombus on both the outflow and inflow. The thrombus and aortic valve were carefully excised and the annulus debrided. A 23 mm St. Jude Medical Regent Mechanical Heart Valve (St. Jude Medical, Inc.) was selected. Multiple 2-0 ETHIBOND EXCEL^®^ sutures (Ethicon US, LLC) without pledgets were placed around the annulus and used to seat the bioprosthesis in a supra-annular fashion. The aortotomy was closed and the patient was weaned off CPB. Intraoperative echocardiography showed a well-seated valve with a mean gradient of 8 mm Hg and no perivalvular leak (Video 1). The patient was extubated the next day. Intraoperatively, the resected valve was sent to pathology, where tissue culture was positive for *S. epidermidis*. In addition, hematologic workup was significant for elevated lupus anticoagulant, elevated beta-2 glycoprotein, and elevated cardiolipin antibodies, indicating high titer triple-positive antiphospholipid syndrome. Figure 1 demonstrates the intraoperative and pathology specimen findings. Infectious disease was consulted and agreed that the most likely scenario appeared to be marantic endocarditis with some degree of superinfection with *S. epidermidis*. The patient was treated for endocarditis and underwent daptomycin treatment for 6 weeks. He was discharged on anticoagulation therapy with a target international normalized ratio of 3.0 to 3.5 due to his hypercoagulable state.

**Video 1:** Marantic endocarditis echocardiogram, also at https://youtu.be/akg4UiTxh4M.

Nonbacterial thrombotic endocarditis (NBTE) is characterized by deposition of sterile platelet thrombi and fibrin on cardiac valves, most commonly the aortic and mitral.^1^ Common causes of NBTE include malignancy, autoimmune diseases such as systemic lupus erythematosus (SLE), and APS. The pathogenesis of marantic endocarditis involves endothelial damage that triggers platelet and fibrin deposition, especially in the presence of a previously activated coagulation system, as in APS.

Compared to vegetations in infective endocarditis, vegetations in NBTE are easily dislodged since there is little inflammatory reaction at the site of the attachment. Thus, there is a greater tendency for vegetations to embolize and cause extensive infarction due to systemic emboli in up to 50% of patients, presenting as flank pain, hematuria, rash, and digital ischemia.^2^ Additionally, embolization may occur in the central nervous system, leading to stroke and delirium, as well as the coronary arteries, leading to chest pain. Despite his superinfection, our patient presented with no symptoms of systemic emboli but, rather, with aortic stenosis, which is an uncommon presentation of NBTE.^2,3^

## Consent

The authors attest they are in compliance with human studies committees and animal welfare regulations of the authors’ institutions and US Food and Drug Administration guidelines, including patient consent where appropriate.

## References

[1] StatPearls [Internet]. Treasure Island, FL: StatPearls Publishing; 2022. Abdisamad M, Ibrahim AM, Siddique MS. StatPearls: Libman Sacks Endocarditis; 2022 May 15 [cited 2022 Jun 15]. Available from: https://www.ncbi.nlm.nih.gov/books/NBK532864/

[2] Sanjay A, Anish P, Omar AK, Rajan S, Sunil KO. Non-bacterial thrombotic endocarditis. Eur J Cardiothorac Surg. 2007 Nov;32(5):696–701. doi: 10.1016/j.ejcts.2007.07.029 17881239

[3] Kurdi M, Beanlands DS, Chan KL, Veinot JP. Nonbacterial thrombotic endocarditis presenting as aortic stenosis with suspected infective endocarditis: clinicopathological correlation. Can J Cardiol. 2004 Apr;20(5):549–552. PMID: 15100758 15100758

